# Hesperidin Protects SH−SY5Y Neuronal Cells against High Glucose−Induced Apoptosis via Regulation of MAPK Signaling

**DOI:** 10.3390/antiox11091707

**Published:** 2022-08-30

**Authors:** Chaemoon Lim, Ao Xuan Zhen, Sungwoo Ok, Pincha Devage Sameera Madushan Fernando, Herath Mudiyanselage Udari Lakmini Herath, Mei Jing Piao, Kyoung Ah Kang, Jin Won Hyun

**Affiliations:** 1 Department of Orthopedic Surgery, Jeju National University Hospital, College of Medicine, Jeju National University, Jeju 63241, Korea; 2 Department of Biochemistry, College of Medicine, Jeju National University, Jeju 63243, Korea; 3 Jeju Research Center for Natural Medicine, Jeju National University, Jeju 63243, Korea

**Keywords:** hesperidin, glucose, SH−SY5Y neuronal cell, oxidative stress, apoptosis

## Abstract

Neurodegenerative diseases are associated with neuronal cell death through apoptosis. Apoptosis is tightly associated with the overproduction of reactive oxygen species (ROS), and high glucose levels contribute to higher oxidative stress in diabetic patients. Hesperidin, a natural active compound, has been reported to scavenge free radicals. Only a few studies have explored the protective effects of hesperidin against high glucose−induced apoptosis in SH−SY5Y neuronal cells. Glucose stimulated neuronal cells to generate excessive ROS and caused DNA damage. In addition, glucose triggered endoplasmic reticulum stress and upregulated cytoplasmic as well as mitochondrial calcium levels. Hesperidin inhibited glucose−induced ROS production and mitigated the associated DNA damage and endoplasmic reticulum stress. The downregulation of antiapoptotic protein Bcl−2 following glucose treatment was reversed by a hesperidin treatment. Furthermore, hesperidin repressed the glucose−induced Bcl−2−associated X protein, cleaved caspase−9, and cleaved caspase−3. Hesperidin also suppressed the glucose−induced phosphorylation of extracellular signal−regulated kinase and c−Jun N−terminal kinase. The current results confirmed that hesperidin could protect neuronal cells against glucose−induced ROS. Mechanistically, hesperidin was shown to promote cell viability via attenuation of the mitogen−activated protein kinase signaling pathway.

## 1. Introduction

The prevalence of diabetes mellitus (DM) and its complications are increasing worldwide, which imposes great pressure on patients and medical systems [1]. While neurons have a constant high demand for glucose, diabetic hyperglycemia is known to induce neuronal damage via intracellular glucose metabolism [2]. Diabetic neuropathy is the most frequent complication of DM and the most important reason for foot ulcers as well as nontraumatic amputations [3]. Patients with diabetic neuropathy have a compromised quality of life due to pain, disability, and hospitalization [4]. Further, the cost of treatment for diabetic neuropathy increases annually, in parallel to DM incidence and prevalence [1]. In recent years, many researchers have worked to elucidate the pathogenesis of diabetic neuropathy and develop novel treatments based on the pathogenic mechanism.

Hyperglycemia is an established factor that induces oxidative stress and endoplasmic reticulum (ER) stress, leading to extensive injury in the peripheral nervous system with various types of cells (such as Schwann cells, sensory neurons, and myelinated axons) [5,6]. Among these, oxidative stress−induced damage within the mitochondria−rich axons drives their degeneration or neuronal apoptosis [7]. Oxidative stress−induced apoptosis plays a central role in several neurodegenerative diseases, such as Huntington’s, Alzheimer’s, and Parkinson’s diseases [8]. Similarly, calcium homeostasis and associated mitochondrial dysfunction play an important role in the pathogenesis of diabetes [9]. One study has suggested that increasing the level of antioxidant enzymes in mitochondria, such as glutathione, may be a therapeutic strategy for neurodegenerative disorders caused by oxidative stress, and can be assessed by mitochondrial membrane potential and cytochrome c release, among other techniques [10].

Flavonoids are a group of natural hydroxylated phenolic compounds found in citrus fruits, vegetables, and tea [11]. Their free radical scavenging capacity makes flavonoids potential therapeutic compounds against oxidative stress−driven disease [12,13]. Further, the antioxidant effect of flavonoids in diabetes has been reported in many studies [14,15]. Recently, hesperidin, a flavanone glycoside, has been identified as a neuroprotective compound against Parkinson’s disease or Alzheimer’s disease [16,17]. Clinical and in vivo studies have shown that hesperidin protects against neurodegeneration via the upregulation of intracellular antioxidant defenses [18]. The SH−SY5Y neuroblastoma cell line is most widely used for in vitro studies of Parkinson’s disease [19]. As the protective mechanism of hesperidin against high glucose−induced neuronal cell damage remains unclear, we examined its antioxidant and cytoprotective effects against hyperglycemia−induced apoptosis in SH−SY5Y cells as well as the underlying mechanism of action.

## 2. Materials and Methods

### 2.1. Reagents and Chemicals

**Figure 1:**
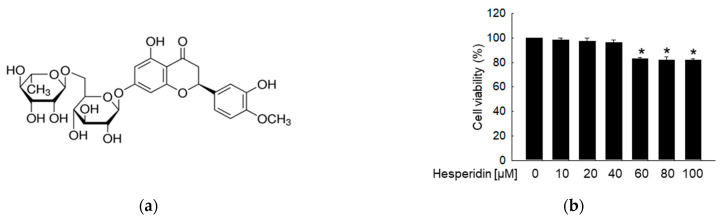

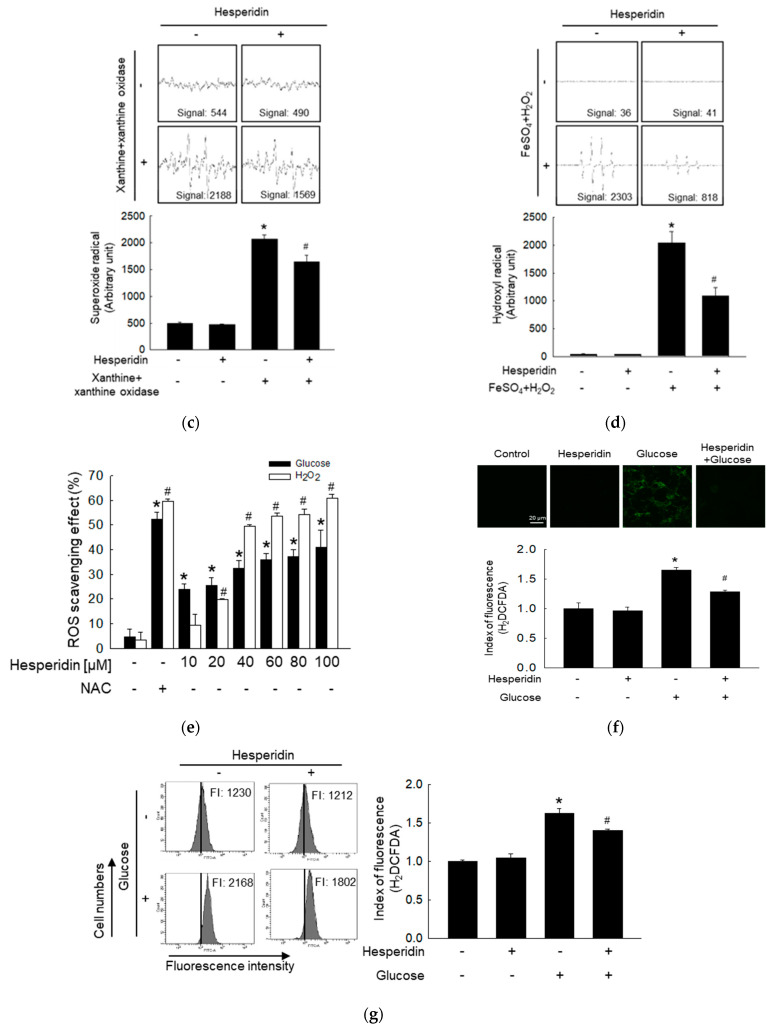
Hesperidin protected SH−SY5Y neuronal cells against high glucose−induced oxidative stress. (**a**) Chemical structure of hesperidin. (**b**) Cell viability was assessed using the MTT assay. * *p* < 0.05 compared to control cells. (**c**) The superoxide radical was examined via ESR. * *p* < 0.05, # *p* < 0.05 compared to control and xanthine + xanthine oxidase, respectively. (**d**) The hydroxyl radical was examined via ESR. * *p* < 0.05, # *p* < 0.05 compared to control and FeSO_4_ + H_2_O_2_, respectively. (**e**) The ROS were determined using spectrometry after H_2_DCFDA staining. * *p* < 0.05, # *p* < 0.05 compared to glucose− and H_2_O_2_−exposed cells, respectively. (**f**,**g**) The ROS were analyzed using confocal microscopy and flow cytometry after H_2_DCFDA staining. * *p* < 0.05, # *p* < 0.05 compared to control cells and glucose−exposed cells, respectively.

Hesperidin (Figure 1a), glucose, N−acetylcysteine (NAC), 2′,7′−dichlorodihydrofluorescein diacetate (H_2_DCFDA), avidin−tetramethyl−rhodamine isothiocyanate (TRITC) conjugate, and actin were obtained from Sigma−Aldrich (St. Louis, MO, USA). 5,5−Dimethyl−1−pyrroline−N−oxide (DMPO) was purchased from Cayman Chemical (Ann Arbor, MI, USA). Fluo−4 acetoxymethyl ester (Fluo−4 AM) and Rhod−2 AM were purchased from Molecular Probes (Eugene, OR, USA). Hoechst 33342 was provided by Biomol GmbH (Hamburg, Germany). Inhibitors of U0126 and SP600125 were provided from Merck KGaA (Darmstadt, Germany) and Tocris (Ellisville, MO, USA), respectively. Primary antibodies against phospho−H2A.X (Ser139), C/EBP homologous protein (CHOP), caspase−9, caspase−3, phospho−extracellular signal−regulated kinase (ERK), phospho−c−Jun N−terminal kinase (JNK), and JNK were purchased from Cell Signaling Technology (Beverly, MA, USA). Primary antibodies against Bcl−2, Bcl−2−associated X protein (Bax), phospho−protein kinase R−like ER kinase (PERK), phospho−eukaryotic initiation factor 2 alpha (eIF2α), and ERK2 were purchased from Santa Cruz Biotechnology (Dallas, TX, USA). Anti−IgG secondary antibodies were purchased from Invitrogen (Rockford, IL, USA). All other chemicals and reagents used were of analytical grade.

### 2.2. Cell Culture

SH−SY5Y neuronal cells were seeded in Dulbecco’s modified Eagle’s medium supplemented with 10% fetal bovine serum and 1% antibiotic−antimycotic (Life Technologies Co., Grand Island, NY, USA). Cells were cultured at 37 °C in a humidified atmosphere containing 5% CO_2_. For assaying the effects of high glucose on SH−SY5Y cells, cells were cultured in the above medium containing 50 mM glucose with or without hesperidin.

### 2.3. Cell Viability

The 3−(4,5−dimethylthiazol−2−yl)−2,5−diphenyltetrazolium bromide (MTT; Sigma−Aldrich) assay was performed to test the cytotoxic effect of hesperidin on cells, which was determined by detecting the mitochondrial dehydrogenase−mediated cleavage of the tetrazolium salt in viable cells [20]. Cells were cultured in a 24−well plate at a concentration of 1 × 10^5^ cells/mL for 16 h. The cells in the medium with or without glucose at 50 mM were treated with hesperidin at different concentrations, U0126, or SP600125 for 3 days. MTT stock solution was added for 4 h to yield formazan crystals and dissolved in dimethyl sulfoxide. Finally, the absorbance was measured at 540 nm using a scanning multi−well spectrophotometer (Sunrise, Tecan, Maennedorf, Switzerland).

### 2.4. Analysis of Superoxide and Hydroxyl Radicals

Superoxide radicals were generated by xanthine and xanthine oxidase, and hydroxyl radicals were generated via the Fenton reaction (FeSO_4_ + H_2_O_2_). The DMPO signals of O_2_^−^ and OH⋅ were detected using a JES−FA200 electron spin resonance (ESR) spectrometer (JEOL Ltd., Tokyo, Japan), with the parameters set as previously described [21].

### 2.5. Intracellular Reactive Oxygen Species (ROS) Measurement

The ability of hesperidin to inhibit high glucose−induced intracellular ROS production was investigated using H_2_DCFDA. Cells were seeded in a 96−well plate at 1 × 10^5^ cells/mL and treated with hesperidin (10, 20, 40, 60, 80, or 100 μM) or NAC (1 mM) for 16 h after seeding. After 30 min, 50 mM glucose was added to each well. The cells were cultured for an additional 30 min and the fluorescence of H_2_DCFDA was detected using a GloMax^®^−Multi Base Instrument (Promega, Madison, WI, USA). Similarly, cells stained with H_2_DCFDA were detected via FV1200 confocal microscopy (Olympus, Tokyo, Japan) and flow cytometry (Becton Dickinson, Mountain View, CA, USA) [22].

### 2.6. 8−Oxoguanine (8−oxoG) Detection

Cells were cultured under high glucose in a chamber slide at 1.5 × 10^5^ cells/mL. The 8−oxoG modification was observed under a confocal microscope using the avidin−TRITC conjugate [23].

### 2.7. Western Blot Analysis

Cells were exposed to 50 mM glucose on different days or 20 μM hesperidin and 50 mM glucose simultaneously for three days. Total protein was obtained from the harvested cells and separated via SDS−PAGE, where proteins were transferred to the membrane. The membranes were incubated with primary antibodies against phospho−H2A.X, phospho−PERK, phospho−eIF2α, CHOP, Bcl−2, Bax, caspase−9, caspase−3, phospho−ERK, ERK2, phospho−JNK, JNK, and actin, followed by incubation with secondary antibodies for another 1 h. Bands were visualized using an Amersham ECL western blotting detection reagent (Cytiva, Buckinghamshire, UK).

### 2.8. Cellular and Mitochondrial Calcium Level Detection

As previously described, we used Fluo−4 AM and Rhod−2 AM to detect total calcium and mitochondrial calcium levels in neuronal cells, respectively [24]. Cells were seeded into 6−well plates and incubated with Fluo−4 AM or Rhod−2 AM for 30 min. The fluorescence of intracellular and mitochondrial Ca^2+^ was measured using flow cytometry [25].

### 2.9. Hoechst 33342 Staining

Hoechst 33342, a nuclear−specific dye, was used to evaluate apoptotic bodies to stain SH−SY5Y cell nuclei. Cells were cotreated with hesperidin and glucose for 3 days. Hoechst 33342 was then added to each group, which was further maintained for 10 min in the dark. To observe the nuclear condensation, apoptotic nuclei were observed under an Olympus 100W Mercury Power Supply (Olympus, Tokyo, Japan) [26].

### 2.10. Statistical Analysis

All experiments were performed in triplicates. Data are presented as the mean ± standard error. Means were compared using an analysis of variance (ANOVA), followed by Tukey’s test. *p* < 0.05 was considered significant.

## 3. Results

### 3.1. Hesperidin Protects Neuronal Cells from High Glucose−Induced Oxidative Stress

In the MTT assay, hesperidin showed no cytotoxicity at doses below 40 μM (Figure 1b). During glucose metabolism within mitochondria, ROS induced by high glucose was assessed via superoxide and hydroxyl radicals [27]. The superoxide radical scavenging ability of hesperidin was detected at 20 μM, with significant suppression of superoxide radical levels (Figure 1c). Similarly, 20 μM hesperidin significantly reduced hydroxyl radicals (Figure 1d). We then confirmed the intracellular ROS scavenging ability of hesperidin in SH−SY5Y neuronal cells, as high glucose levels, like H_2_O_2_, induced high levels of ROS, which were inhibited by hesperidin in a dose−dependent manner (Figure 1e). Based on results from the cell viability, ESR, and ROS scavenging tests, 20 μM hesperidin was found to be the optimal concentration in this study. According to a previous study on high glucose−induced cytotoxicity and apoptosis, glucose at 50 mM significantly inhibited the cell viability of SH−SY5Y and induced apoptosis after 2 days [28]. Thus, 50 mM was set as the high glucose concentration in our assays. To confirm the ROS scavenging ability of hesperidin, we subjected cells to confocal microscopy and flow cytometry analyses. Both results indicated that the high levels of ROS induced by glucose in SH−SY5Y cells were inhibited under hesperidin treatment (Figure 1f,g). Taken together, hesperidin protected neuronal cells from high glucose−induced ROS, and we selected 20 μM as the optimal concentration for subsequent cellular experiments.

### 3.2. Hesperidin Ameliorated Glucose−Induced DNA Damage

**Figure 2:**
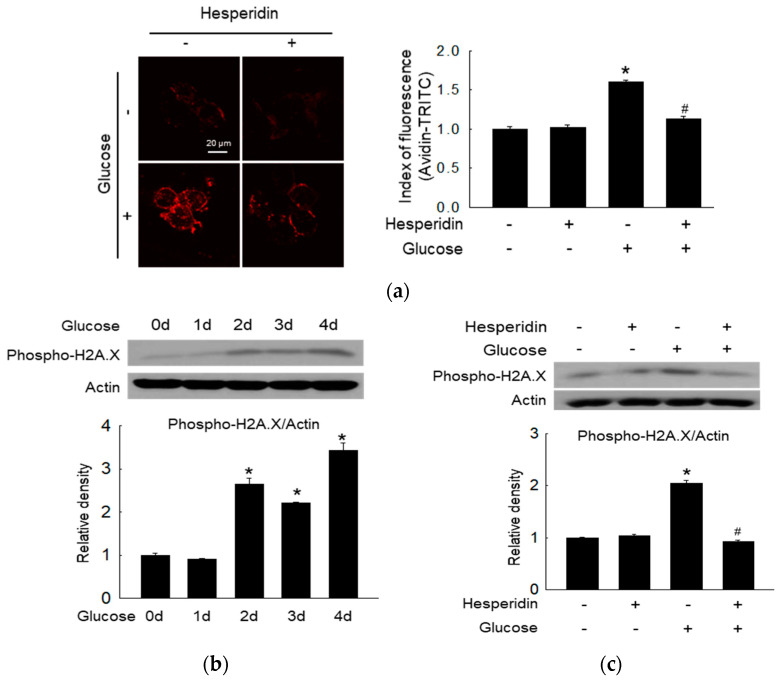
Hesperidin relieved high glucose−induced DNA damage in SH−SY5Y cells. (**a**) 8−OxoG was assessed using confocal microscopy after staining of avidin−TRITC. (**b**,**c**) Phospho−H2A.X was measured via western blotting. Actin was regarded as a loading control. The ‘d’ stands for ‘day’ after cells were treated with glucose. * *p* < 0.05 and # *p* < 0.05 compared to control cells and glucose−exposed cells, respectively.

Oxidative stress promotes neurodegenerative disease via the disruption of the DNA structure [29]. Glucose metabolism is associated with increased levels of 8−oxoG in cells, which hesperidin alleviated (Figure 2a). The phosphorylation of histone H2A.X, a specific DNA damage marker, was induced by glucose treatment in a time−dependent manner (Figure 2b). However, hesperidin reversed the glucose−induced upregulation of phospho−H2A.X (Figure 2c). Thus, hesperidin protected neuronal cells from glucose−induced DNA damage.

### 3.3. Hesperidin Suppressed Glucose−Induced ER Stress Response

**Figure 3:**
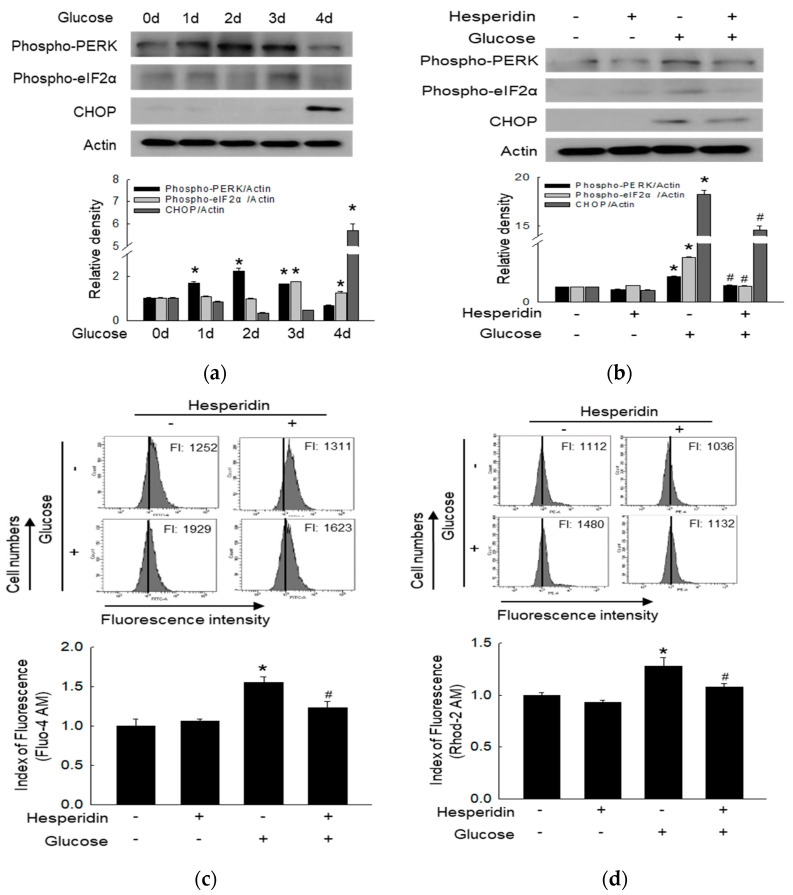
Hesperidin mitigated high glucose−induced ER stress in SH−SY5Y neuronal cells. (**a**,**b**) Phospho−PERK, phospho−eIF2α, and CHOP were detected via western blot. (**c**) ER calcium levels were analyzed by Fluo−4 AM dye. (**d**) Mitochondrial calcium levels were measured by Rhod−2 AM dye. * *p* < 0.05 and # *p* < 0.05 compared to control cells and glucose−exposed cells, respectively.

ROS can also promote neuronal injury via ER stress [30]. Glucose upregulated the protein levels of phospho−PERK, phospho−eIF2α, and CHOP over time, which belong to the ER stress (Figure 3a). Hesperidin suppressed these ER stress-associated factors (Figure 3b). Cytoplasmic calcium levels were upregulated under treatment with glucose, which was once again reversed through hesperidin pretreatment (Figure 3c). Furthermore, hesperidin restored mitochondrial calcium homeostasis (Figure 3d). Taken together, hesperidin suppressed high glucose−induced ER stress and maintained cellular calcium levels.

### 3.4. Hesperidin Protected Neuronal Cells against Glucose−Induced Apoptosis

**Figure 4:**
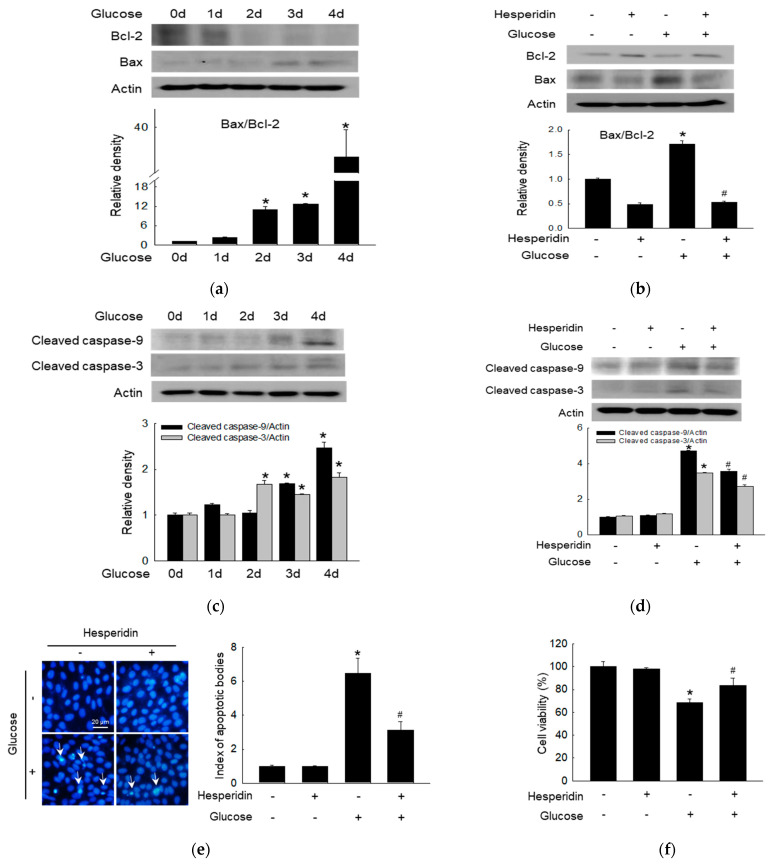
Hesperidin protected SH−SY5Y neuronal cells against high glucose−induced apoptosis. (**a,b**) Bcl−2 and Bax were detected via western blot. (**c**,**d**) Cleaved caspase−9 and cleaved caspase−3 were detected via western blot. (**e**) Hoechst 33342 dye was used for the observation of apoptotic bodies. (**f**) Cell viability was detected via MTT assays. * *p* < 0.05 and # *p* < 0.05 compared to control cells and glucose−exposed cells, respectively.

The Bcl−2 was suppressed, while Bax was upregulated by glucose in a time−dependent manner, which represented antiapoptotic proteins and proapoptotic proteins, respectively (Figure 4a). However, hesperidin treatment reversed these expression changes (Figure 4b). Apoptosis effectors caspase−9 and caspase−3 were activated by glucose over time (Figure 4c), which was suppressed by the treatment with hesperidin (Figure 4d). The inhibition apoptotic effect of hesperidin was confirmed through the observation of glucose−induced apoptotic bodies via Hoechst 33342 staining, which was reversed by hesperidin (Figure 4e). Finally, glucose suppressed cell viability, once again reversed by hesperidin treatment (Figure 4f). Taken together, hesperidin suppressed glucose−induced neuronal apoptosis.

### 3.5. Hesperidin Protected Neuronal Cells via Attenuation of ERK/JNK Signaling Pathway

**Figure 5:**
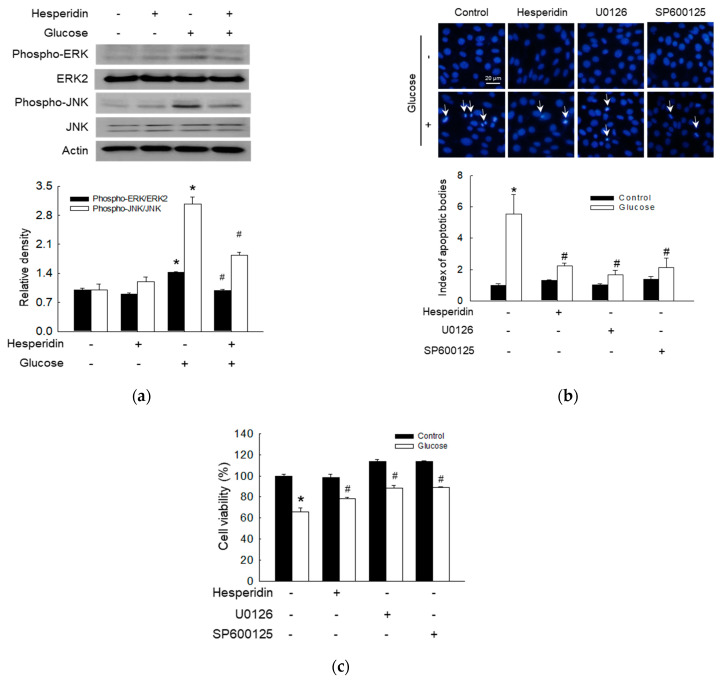
Hesperidin protected SH−SY5Y neuronal cells against high glucose−induced cell death via the ERK and JNK signaling pathways. (**a**) Phospho−ERK, ERK2, phospho−JNK, and JNK were measured via western blot. (**b**) Apoptotic bodies were detected using Hoechst 33342. Arrow indicates apoptotic body. (**c**) Cell viability was detected via MTT assay. * *p* < 0.05 and # *p* < 0.05 compared to control cells and glucose−exposed cells, respectively.

A recent study reported the activation of mitogen−activated protein kinases (MAPKs) in SH−SY5Y cells undergoing H_2_O_2_−induced apoptosis [31]. Hesperidin suppressed phospho−ERK and phospho−JNK, which were stimulated by glucose (Figure 5a). Cells cultured in the presence of glucose as well as mitogen−activated protein kinase/ERK kinase (MEK, the upstream of ERK) [31] and JNK inhibitors (U0126 and SP600125, respectively) exhibited a lower index of apoptotic bodies than those in the glucose alone group, but similar to that in the hesperidin and glucose group (Figure 5b). After treatment with MEK and JNK inhibitors (U0126 and SP600125, respectively), the glucose−induced reduction in cell viability was enhanced, yet treatment with hesperidin reversed it (Figure 5c). Taken together, hesperidin protected against apoptosis and promoted cell viability via inhibition of the ERK and JNK MAPK signaling pathways.

## 4. Discussion

Hyperglycemia−induced oxidative stress is a critical mechanism in the pathogenesis of diabetic neuropathy. A previous study revealed that high glucose inhibited neural cell differentiation via oxidative stress and ER stress, causing neural tube defects in maternal diabetic patients [32]. Increased oxidative stress has an adverse effect on the metabolism of the peripheral nervous system [33], promoting axon degeneration in the early stage of diabetes [34]. Interestingly, increasing doses of free radical accumulation stimulate the hermetic response (a low dose stimulation and a high dose inhibition) of the intrinsic cellular redox homeostasis system (including free radical damage and decreased energy production), resulting in neurodegeneration during the aging process, and notably, this dose−response relationship may modulate the neuroprotective effects [35,36]. Hormesis provides a novel insight into assessing the therapeutic potentiality of pharmaceutical drug candidates based on their neuroprotective abilities. Herein, to explore new treatment strategies for neurodegenerative diseases, we assessed hesperidin known for its antioxidative effects. Hesperidin has diverse biological activities, including antioxidative and anti−inflammatory effects, mediated via radical scavenging and the promotion of antioxidant defense [37]. Hesperidin was shown to alleviate retinal and plasma abnormalities in diabetic rats via inhibition of ROS [38]. In the present study, SH−SY5Y cells were cultured under high glucose to mimic nerve cells’ response to high glucose. High glucose upregulated intracellular ROS, which were significantly reduced by hesperidin treatment. In addition, ROS can trigger the cellular DNA damage response, which is intimately linked to the manifestation of neurodegenerative disorders [39]. Hesperidin was shown to modulate the high glucose−induced DNA damage response. These properties of hesperidin are expected to be beneficial against diabetic neuropathy.

Increased ROS and compromised endogenous antioxidant defense can disturb protein folding, leading to the accumulation of unfolded proteins [40]. These in turn activate PERK localized on the ER membrane by triggering its homodimerization and autophosphorylation. PERK activation leads to eIF2α phosphorylation, which in turn promotes CHOP expression. CHOP then activates the action of cytochrome c release and expression of proapoptotic proteins, such as Bax, while inhibiting antiapoptotic proteins, such as Bcl−2 [41]. Herein, we confirmed that a high glucose level upregulated the phosphorylation of PERK and eIF2α as well as its downstream signaling protein CHOP. However, hesperidin treatment significantly reduced the activation of the PERK/eIF2α/CHOP axis. Cellular calcium overload together with ROS contributes to high levels of mitochondrial calcium, which leads to cell death via mitochondrial dysfunction, observed in several neural diseases [42]. The upregulation of mitochondrial glutathione can prevent neurons from free radicals (such as nitric oxide) induced damage and has important implications for liver diseases and neurodegenerative disorders [10,43]. Moreover, the mitochondrial membrane potential is a determinant of cell apoptosis and calcium homeostasis [44]. Our results showed that hesperidin could restore calcium homeostasis, which may be beneficial for inhibiting ER stress−induced diabetic neuropathy.

High glucose−induced ROS can activate Bax and inhibit Bcl−2, triggering cell apoptosis [45]. The apoptosome complex can cleave caspase−3, one of the effector caspases, leading to the degradation of cellular proteins and subsequent apoptosis [46]. Moreover, increased cleavage of caspase−9 and caspase−3 has been detected in neurons and Schwann cells from 12−month diabetic rats under high glucose [47]. In addition, hesperidin inhibited high glucose−induced apoptosis by regulating mitochondria−related proteins, including the reduction of Bcl−2 expression, induction of Bax expression, as well as the cleavage of caspase−9 and −3 in SH−SY5Y neuronal cells. Thus, hesperidin has the potential to suppress neuronal cell death.

The MAPK pathway has been reported to participate in cell proliferation, senescence, and apoptosis [48]. The other two studies also showed that a high glucose level triggered DNA damage response and dysregulated MAPK via excessive ROS in hyperglycemia, and an unfolded protein response in ER stress could regulate phosphorylation of JNK causing apoptosis [5,49]. In this study, a high glucose level significantly increased the levels of phospho−ERK and phospho−JNK, indicating that the ERK and JNK pathways were activated. However, hesperidin treatment significantly reduced both levels. Furthermore, hesperidin, an ERK inhibitor, and a JNK inhibitor increased cell viability by suppressing apoptosis, indicating that hesperidin protects neural cells from glucose−induced cytotoxicity via attenuation of the MAPK signaling pathway.

## 5. Conclusions

**Figure 6:**
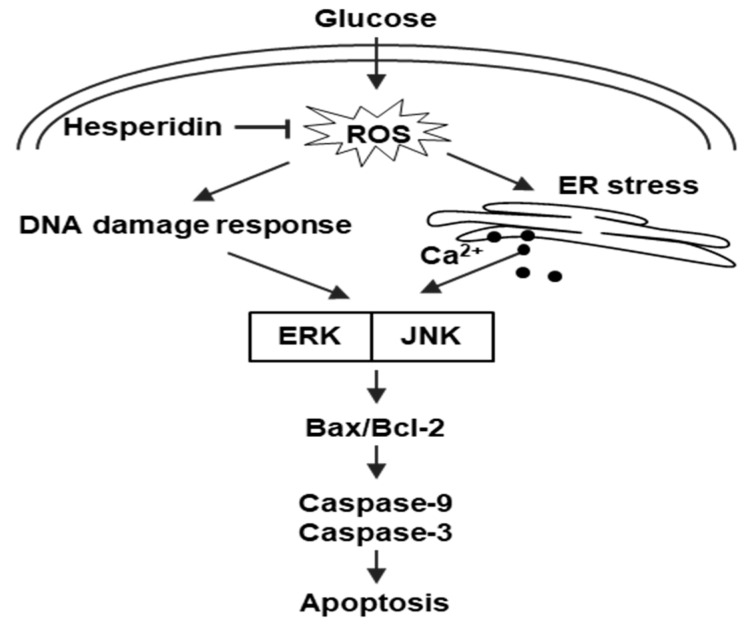
Schematic diagram of hesperidin’s protective effects against high glucose−induced SH−SY5Y neuronal cell death. Glucose−induced ROS in neuronal cells cause DNA damage and ER stress, in turn triggering apoptosis. However, hesperidin suppresses ROS levels and protects cells from apoptosis through inhibition of the MAPK signaling pathway.

In conclusion, hesperidin markedly inhibited high glucose−induced ROS production through its antioxidant effect in SH−SY5Y neuronal cells (Figure 6). By scavenging ROS, hesperidin effectively protected SH−SY5Y neuronal cells against oxidative injury, ER stress, and apoptosis. And hesperidin inhibited the activation of ERK and JNK, which induced by oxidative stress, and recovered MAPK signaling-reduced cell viability. Our study suggests that hesperidin is a promising biomolecular for diabetic neuropathy treatment.

## Data Availability

The data presented in this study are available on request from the corresponding author.
